# Comparative Assessment of Relapse Following Fixed Orthodontic Treatment in Patients Treated With and Without Extraction: A Systematic Review and Meta-Analysis

**DOI:** 10.7759/cureus.79990

**Published:** 2025-03-03

**Authors:** Priya Rajput, Suryakant Powar, Sumeet Ghonmode, Pallavi G Chaudhary, Anisha Rajan CM

**Affiliations:** 1 Orthodontics and Dentofacial Orthopaedics, Government Dental College and Hospital, Mumbai, IND

**Keywords:** extraction vs non-extraction, incisor crowding, meta-analysis, orthodontic relapse, overbite, overjet, systematic review

## Abstract

The present systematic review aimed to evaluate and compare the post-treatment relapse of overjet, overbite, and mandibular incisor crowding in patients treated with extraction and non-extraction orthodontic approaches and to assess the influence of treatment modality on long-term stability. The review was conducted following PRISMA guidelines. Data were extracted from 10 studies, including retrospective and longitudinal designs, with a total of 720 participants. The primary outcome measures included overjet, overbite, and Little's Irregularity Index. Standardized mean difference (SMD) was used as the summary statistic to compare extraction and non-extraction groups. The risk of bias was assessed using the ROBINS-I tool. The meta-analysis revealed no statistically significant differences in relapse between extraction and non-extraction groups for overjet (SMD: 0.52, 95% CI: -0.18 to 1.23, p>0.05), overbite (SMD: 0.41, 95% CI: -0.11 to 0.93, p>0.05), and incisor irregularity index (SMD: 0.71, 95% CI: -0 to 1.23, p>0.05). Funnel plots indicated no significant publication bias. Risk of bias assessment showed moderate concerns in confounding and participant selection across several studies, while bias due to missing data was notable in longer follow-up studies. The findings suggest that both extraction and non-extraction treatment approaches exhibit similar post-treatment relapse rates for overjet, overbite, and incisor irregularity. The choice of treatment should be based on clinical considerations along with concerns of long-term stability. Further high-quality randomized controlled trials are required to strengthen the evidence.

## Introduction and background

Dental extractions have been an inseparable part of orthodontic practice in cases where space gain is required, with premolars being the most commonly extracted teeth. However, the issue has been a matter of debate with many reports in favor of non-extraction orthodontic treatment. The practitioners supporting this notion believe that the extraction of premolars can have a more detrimental effect on the patient’s facial development than the actual malocclusion [1]. Additionally, while malocclusion is corrected during orthodontic treatment, there is alarming clinical evidence of post-treatment relapse, which is cause for concern [2,3].

The essence of orthodontic treatment lies in correcting the malocclusions present in the dentition. Equally important is to sustain the treatment outcomes and prevent future recurrence. It is universally known that relapse following orthodontic treatment is inevitable, and some amount of changes in the dentition will occur even after the treatment is completed [2]. Stabilizing the achieved corrections and preventing relapse is a challenge faced by orthodontists. Knowing the factors behind post-treatment relapse can greatly aid orthodontists in the determination of the treatment plan and its prognosis [4,5]. They will be better able to devise strategies to tackle the relapse after treatment.

Extractions followed by fixed orthodontic treatment are usually performed to correct overjet, overbite, and crowding. Some of the factors influencing relapse after overjet correction include the extent of incisal inclination during the treatment commencement, labial inclination of the maxillary incisors, interincisal angle, and lingual inclination of the mandibular incisors [6]. Likewise, factors responsible for relapse after overbite correction include overjet, the distance of displacement of incisors from their natural position, facial pattern and height, crowding, and the amount of overbite corrected [7].

Various systemic factors and the use of necessary systemic medications during and after the orthodontic treatment can influence its post-treatment outcomes and sustainability [8,9]. Because of the numerous factors influencing the post-treatment outcomes leading to relapse, many times, maintaining the achieved corrections is equally, or perhaps, more challenging than the treatment itself. It would cost the patients’ time and money to no benefit, and thus, the patients may lose confidence in the orthodontist when such an unfavorable event occurs. The prevention of relapse, therefore, starts at the time of treatment planning, even before the treatment has begun. Occlusal harmony, tooth-soft tissue relationships, space gain, and other such factors need to be meticulously considered before initiating a fixed orthodontic treatment plan [10,11]. In this regard, answering the question of whether gaining space by dental extractions is more beneficial compared to its collateral effects can play a pivotal role in deciding the treatment plan by the orthodontists.

Determining whether space gained through extractions provides greater long-term stability compared to its potential drawbacks remains a crucial consideration in orthodontic decision-making. Existing clinical literature presents conflicting evidence regarding the impact of extractions on post-treatment relapse. While some studies suggest that extraction therapy contributes to better long-term stability, others report no significant differences between extraction and non-extraction approaches. Previous systematic reviews have attempted to address this issue, yet variability in study designs, follow-up durations, and relapse assessment methods has led to inconclusive findings. To clarify this controversy, the present systematic review aims to compare the extent and rate of relapse following the correction of overjet, overbite, and crowding with and without extractions in fixed orthodontic treatment. By synthesizing high-quality evidence, this review seeks to provide orthodontists with a clearer understanding of whether extraction or non-extraction treatment protocols offer superior long-term stability.

## Review

Protocol development

The present systematic review was conducted and performed according to the preferred reporting items for systematic review and meta-analysis (PRISMA) statement [12]. The review protocol was registered in Prospective Registration of Systematic Review (PROSPERO) - CRD42023485950.

Selection criteria

The focused research question of the review was “Is there any difference in the relapse after fixed orthodontic treatment in patients treated with extraction and without extraction?" The framework behind the research question included patients with malocclusion as the population (P), fixed orthodontic treatment with extraction (I), fixed orthodontic treatment without extraction (C), and relapse as the outcome (O). Randomized and non-randomized clinical trials, observational studies, cross-sectional studies, cohort studies, and retrospective studies were included in the review, whereas case reports/series, animal studies, in vitro studies, and review articles were excluded. Studies published since inception up to December 2023 satisfying the above-mentioned PICO framework with full text available in the English language were included for data extraction. The selection of studies was performed through meticulous screening and mutual discussions by authors (PR, SP, SG, and AR). The author PC was consulted for doubts regarding the eligibility of a study. The final selection was based on consensus among all three authors. The corresponding authors of the study were contacted via email when further information was required.

Search strategy

A comprehensive electronic search was performed till December 2023 for relevant studies in the English language using the following databases: PubMed, Google Scholar, EMBASE, and EBSCOhost. Appropriate keywords and Medical Subject Heading (MeSH) terms were selected and combined with Boolean operators like AND. The relevant data was searched using the following keywords and their combinations: “extraction” (MeSH term) AND “orthodontics” (MeSH term) AND “crowding” (MeSH term) “overbite” (MeSH term) AND “overjet” (MeSH term) AND malocclusions (MeSH term). Reference lists of the included articles were screened to identify additional articles missed during the search. Grey literature was identified using Google Scholar, Greylist, and OpenGrey.

Data extraction

The data extraction process was carried out systematically to ensure accuracy and consistency across all included studies. A standardized data extraction form was developed to collect relevant information, including study characteristics (author, year, study design, sample size, age group, and gender distribution), intervention details (extraction versus non-extraction groups, type of extractions performed, follow-up duration), and outcome measures (overjet, overbite, and Little's Irregularity Index). Two independent reviewers (PR and AR) extracted data from each eligible study, with discrepancies resolved through discussion or consultation with a third reviewer (SP). Key metrics, such as mean differences and standard deviations, were recorded for both treatment groups. In cases of missing data, the corresponding authors of the respective articles were contacted through e-mail. Additionally, statistical methods used in each study were documented to facilitate further analysis. The extracted data were then synthesized for meta-analysis, ensuring uniformity in reporting and minimizing the risk of bias during data collection.

Assessment of risk of bias, heterogeneity, and quality of evidence

The methodological quality among included studies was executed by using the ROBINS-I tool [13]. The tool comprises seven domains relevant to various types of biases and an overall rating of low, moderate, or high depending on the scores assigned to the individual domains. The significance of any discrepancies in the estimates of the treatment effects of the different trials was assessed by means of Cochran’s test for heterogeneity and the I^2^ statistics. For a value of 0% to 40%, the heterogeneity was considered low; 30 to 60% was considered moderate; 50 to 75% was considered substantial, and greater than 75% was considered high heterogeneity. The certainty of the evidence across all the articles included in the present systematic review was assessed using the GRADE approach [14]. 

Statistical analysis

The standardized mean difference (SDM) with 95% CI was calculated for continuous outcomes. A fixed effects model (Mantel-Haenszel method) was used if there was no heterogeneity (p >0.05 or I-squared ≤24%); otherwise, a random effects model (Der Simonian-Laird method) was used. All statistical analyses were performed using the RevMan 5.3 (Cochrane Collaboration, Software Update, Oxford, UK). The significance level was kept at p<0.05. To test for the presence of publication bias, the relative symmetry of the individual study estimates was assessed around the overall estimates using Begg’s funnel plot. A funnel plot (plot of the effect size versus standard error) was drawn.

Study selection

**Figure 1:**
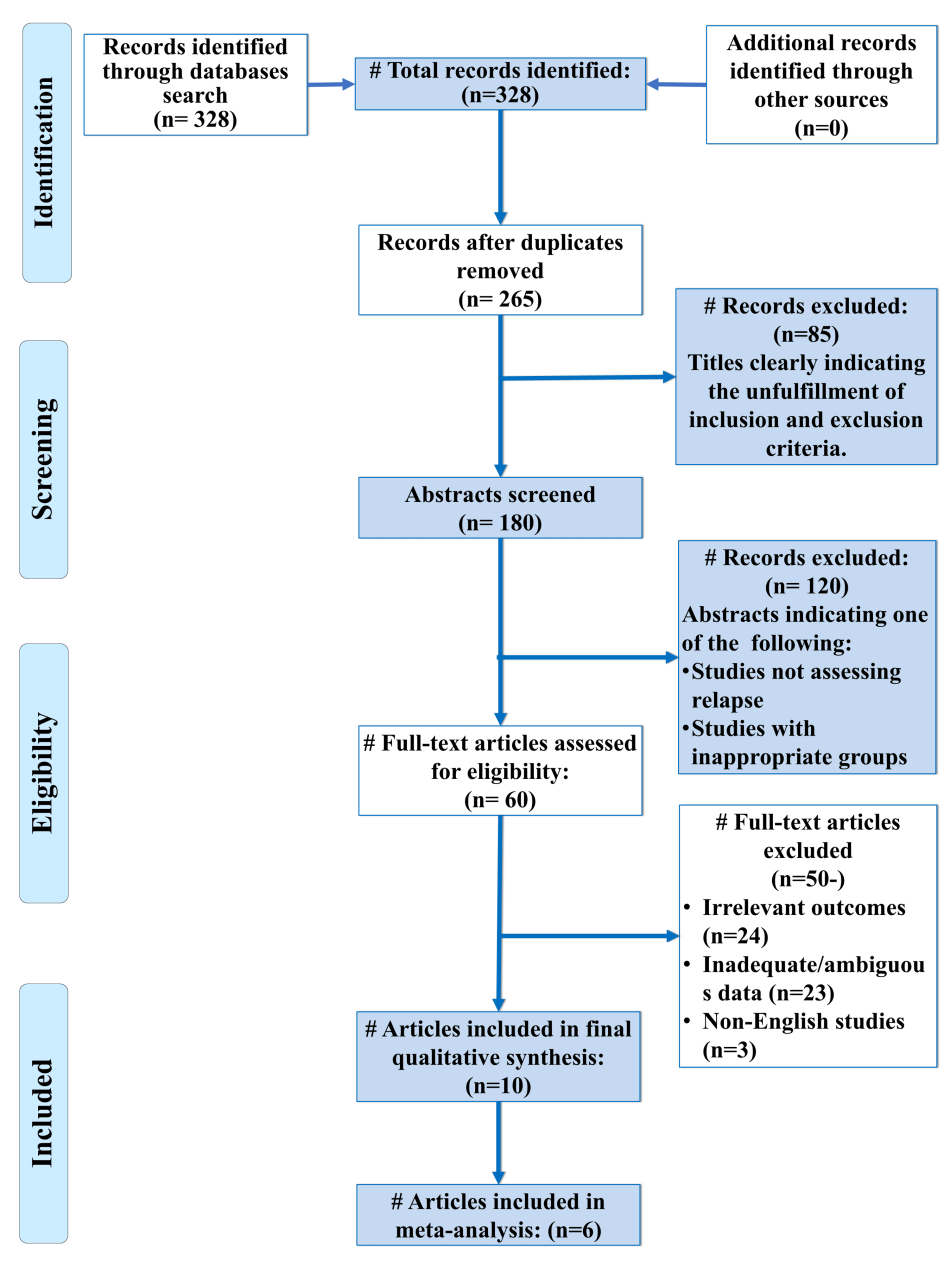
PRISMA flow diagram indicating the selection process of the articles in the present systematic review PRISMA, preferred reporting items for systematic review and meta-analysis

A total of 328 records were identified through database searching. After duplicate removal, the reference list of included studies (n=265) was screened, and 85 studies were excluded. Full-text articles (n=60) were assessed for eligibility, and studies that did not match our inclusion criteria were excluded. Only 10 studies fulfilled eligibility criteria and were included in qualitative synthesis and six studies in meta-analysis [15-24]. A flowchart of identification, inclusion, and exclusion of studies is shown in Figure 1.

Results of the systematic review

A total of 10 studies were included in this systematic review, comprising a diverse range of study designs, including retrospective, longitudinal, and cross-sectional studies. The data extracted from these studies is comprehensively tabulated in Table 1. The sample sizes across the studies ranged from 40 to 120 participants, with age groups spanning from 10 to 38 years. The gender distribution across studies showed a predominance of female participants in most studies, with male-to-female ratios varying across the cohorts.

**Table 1:** Data extracted from the articles included in the present systematic review E, extraction; NE, non-extraction

Author (Year)	Study Design	Sample Size	Age Group (years)	Gender (M/F)	Extraction (N)	Non-extraction (N)	Extraction Details	Follow-Up (Years)	Outcome Measures	Key Metrics	Results (Summary)	Statistical Methods
Hellekant et al. (1989) [15]	Retrospective study	40	Not stated	15/25	20	20	-	2	Overjet, overbite, mandibular intercanine width, maxillary incisor inclination	Overjet relapse: 1.0 mm (NE) vs. 1.4 mm (E); Overbite relapse: 0.8 mm (NE) vs. 1.2 mm (E)	No significant difference in overjet and overbite relapse between groups	ANOVA with repeated measures
Bishara et al. (1994) [16]	Longitudinal study	91	13-18	41/50	45	46	Maxillary first premolars extracted	5	Overjet, overbite, incisor irregularity, arch length	Overjet relapse: 2.0±0.8 mm (E) vs. 2.7±1.1 mm (NE)	Greater incisor irregularity relapse in non-extraction group	Multivariate Analysis, ANOVA
Rossouw et al. (1999) [17]	Longitudinal study	88	10.1-33.5	33/55	39	49	Premolar extractions	6.8	Overjet, overbite, incisor irregularity, arch length, intercanine width, facial height ratios	Lower incisor irregularity: T1-5.1 mm (E) vs. 2.7 mm (NE); T3-1.7 mm (E) vs. 1.7 mm (NE)	No significant post-treatment differences between groups	Wilcoxon, Chi-square, Spearman correlation
Erdinc et al. (2006) [18]	Longitudinal study	98	T1: 12.0±1.5, T2: 14.6±1.8, T3: 20.6±3.2	48/50	49	49	Premolar extractions	6	Irregularity index, intercanine width, arch depth, overbite, overjet	Incisor irregularity relapse: 0.97 mm (E) vs. 0.99 mm (NE)	No significant differences in relapse patterns between groups	Statistical correlation tests
Janson et al. (2012) [19]	Retrospective study	60	Group 1: 12.14 (9.08-15.55), Group 2: 12.87 (10.90-16.62)	31/29	30	30	Maxillary first premolar extractions	Group 1: 7.15 (2.40-15.53), Group 2: 9.25 (3.18-15.32)	Overjet, overbite, molar & canine relationship	Overjet relapse: 0.37 mm (Group 1) vs. 0.55 mm (Group 2); Overbite relapse: 0.98 mm (Group 1) vs. 1.04 mm (Group 2)	No significant difference in stability between extraction and non-extraction	Student t-test, Pearson correlation
Zafarmand et al. (2014) [20]	Retrospective study	40	Non-extraction: 16.3 (T1), 18.9 (T2), 24.7 (T3); Extraction: 14.9 (T1), 17.8 (T2), 24.6 (T3)	Oct-30	21	19	Upper and lower first premolars	6	Mandibular incisor irregularity index	Initial crowding: 7.23 mm (E) vs. 6.13 mm (NE); post-retention crowding: 2.11 mm (E) vs. 1.65 mm (NE)	No statistically significant difference in relapse between groups (P=0.138)	Wilcoxon test, Mann-Whitney test, Friedman test
Francisconi et al. (2014) [21]	Retrospective study	84	Non-extraction: 12.96 (T1), 15.12 (T2), 20.37 (T3); Extraction: 13.01 (T1), 15.16 (T2), 20.61 (T3)	32/52	40	44	Four first premolars extracted	5	Overjet, overbite, maxillary, mandibular incisor crowding	Initial mandibular irregularity: 5.93 mm (NE) vs. 8.06 mm (E); maxillary irregularity relapse: 1.64 mm (NE) vs. 0.89 mm (E)	Greater maxillary crowding relapse in NE group; greater overbite relapse in E group	T-tests, Pearson correlation, chi-square test
Gorucu-Coskuner et al. (2017) [22]	Retrospective study	44	Mean: 13.6 (SD: 3.07)	19/25	15	16	Four first premolar extractions	43.8	Little’s Irregularity Index, intercanine width, interpremolar width, Intermolar width, arch length, arch depth, cephalometric parameters	Irregularity index relapse: extraction (1.96 mm), anterior repositioning splint (ARS) (2.38 mm), non-extraction (3.59 mm); intercanine width decreased in all groups; arch length and depth decreased significantly in extraction group	Significant relapse in all groups, highest in non-extraction group; intercanine width decreased significantly in all groups; lower incisors retroclined in extraction group and proclined in ARS and non-extraction groups	ANOVA, Welch test, repeated measure ANOVA, least significant difference (LSD) post-hoc test, Levene variance homogeneity test
Mahmoudzadeh et al. (2018) [23]	Cross-sectional study	120 (40/group)	15-38 (mean 23.1)	21/99	40	40	Single incisor, premolar extractions, and non-extraction	3.5	Incisor irregularity index, overjet, overbite, arch length, intermolar width, intercanine width	Irregularity index: 0.37±0.31 (non-extraction), 0.37±0.41 (premolar extraction), 0.51±0.47 (incisor extraction)	No significant difference among groups (P=0.2); relapse observed in all groups, significant overjet changes in incisor extraction group	ANOVA, Tukey post hoc, Pearson correlation
Bhupali et al. (2019) [24]	Retrospective cohort study	55	Mean: 15.28	20/35	30	25	Four premolar extractions	3 years	Little's Irregularity Index, intercanine width, ABO (affective, behavioral, and cognitive) Model Grading System	Maxillary incisor irregularity decreased from T0-T1 and increased from T1-T2 (P<0.01); mandibular intercanine width increased at T1 but slightly relapsed at T2	No significant differences in relapse between extraction and non-extraction groups (P>0.05); significant relapse in interproximal contacts (P<0.01)	Mann-Whitney U-test, paired t-test, Wilcoxon signed-rank test

Comparison of extraction versus non-extraction treatments

The included studies assessed the stability of orthodontic treatment outcomes in extraction and non-extraction groups using various outcome measures such as overjet, overbite, incisor irregularity index, intercanine width, arch length, and intermolar width. The studies provided mixed results regarding the impact of extraction on post-treatment stability.

Overjet and Overbite Relapse

Francisconi et al. (2014) reported greater overbite relapse in the extraction group compared to the non-extraction group [21]. Similarly, Hellekant et al. (1989) and Bishara et al. (1994) found no significant differences in overjet relapse between the two groups [15,16]. In contrast, Mahmoudzadeh et al. (2018) reported significant overjet relapse in the incisor extraction group, indicating that extraction of incisors may contribute to greater overjet relapse [23].

Incisor Irregularity Index

Most studies, including Erdinc et al. (2006) and Rossouw et al. (1999), observed no significant differences in incisor irregularity relapse between extraction and non-extraction treatments [17,18]. However, Mahmoudzadeh et al. (2018) and Zafarmand et al. (2014) indicated that mandibular crowding relapse was more pronounced in the non-extraction group compared to extraction-treated individuals [20,23].

Intercanine Width and Arch Length

Studies such as Gorucu-Coskuner et al. (2017) highlighted a significant decrease in intercanine width in all groups, with the highest decrease in the extraction group [22]. Bhupali et al. (2019) also reported a decrease in maxillary arch length post-retention, with similar patterns observed across both groups [24]. A key observation across multiple studies was the tendency of the non-extraction group to exhibit greater arch length reduction, leading to relapse over time.

Follow-up duration and retention protocols

The follow-up periods ranged from two to seven years across studies, with standardized retention protocols commonly involving the use of Hawley or bonded retainers. Studies such as Janson et al. (2012) and Francisconi et al. (2014) indicated that retention protocols significantly influenced post-treatment stability [19,21]. The use of fixed mandibular retainers was associated with improved long-term outcomes in both extraction and non-extraction cases.

Statistical analysis and significance

Various statistical methods were employed across studies, including ANOVA, Wilcoxon tests, Pearson correlation, and t-tests. Most studies reported no statistically significant differences in post-treatment relapse patterns between the extraction and non-extraction groups. However, specific differences in maxillary and mandibular crowding relapse were noted in some studies.

Key findings

Extraction treatment appeared to offer better stability in terms of maxillary incisor crowding and overbite control. Non-extraction cases showed higher relapse rates in terms of incisor irregularity and arch length stability. Retention protocols play a critical role in maintaining treatment outcomes, with fixed retainers showing favorable results in preventing relapse. The findings of this systematic review suggest that both extraction and non-extraction treatment modalities can achieve clinically acceptable stability, with no significant differences in long-term relapse patterns. However, individual variability, retention strategies, and treatment planning play a crucial role in determining post-treatment stability.

Assessment of risk of bias of included studies

**Figure 2:**
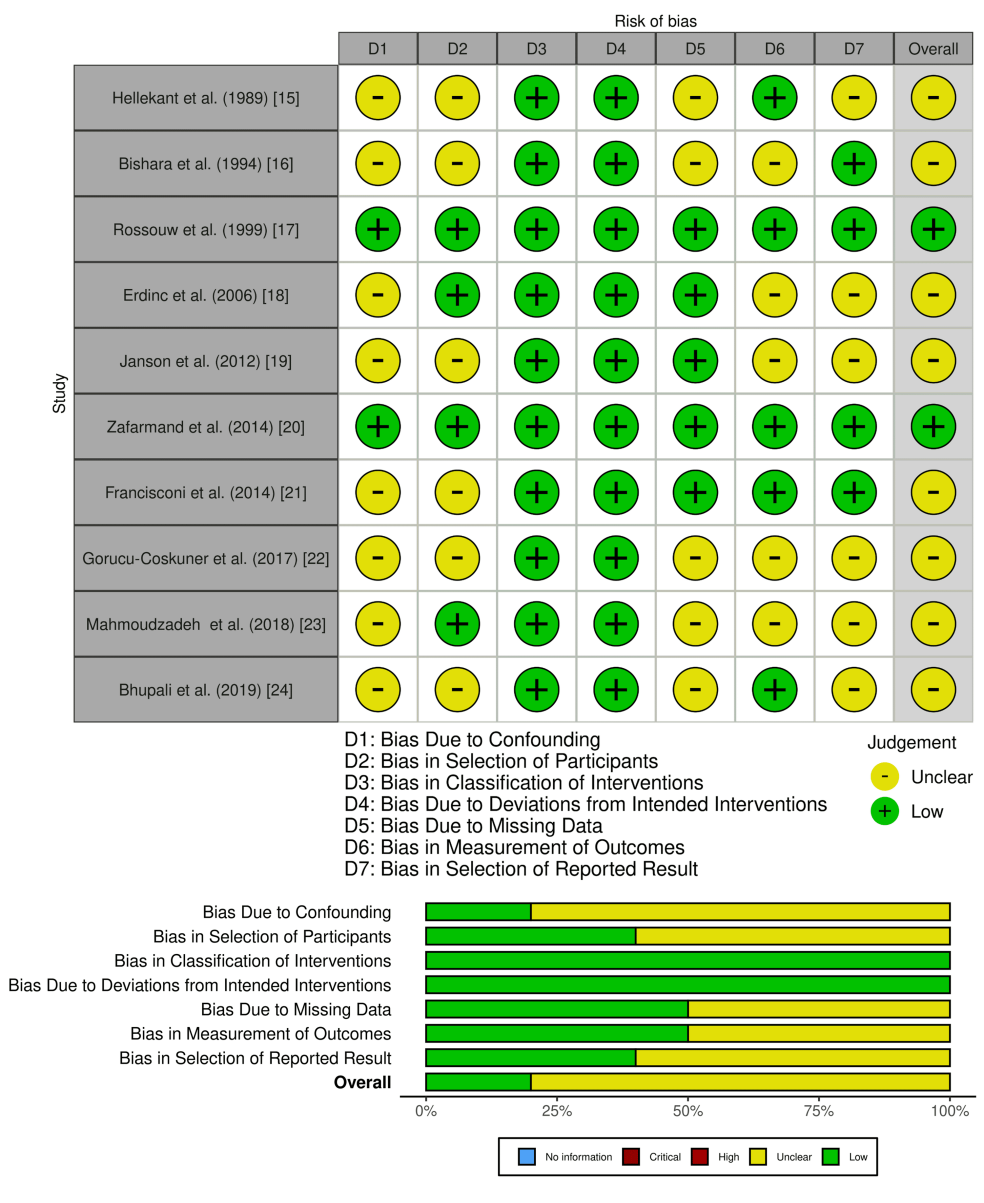
Risk of bias according to the reviewers’ judgments about each domain across all included studies

The studies by Bishara et al. (1994) [16], Rossouw et al. (1999) [17], and Zafarmand et al. (2014) [20] showed a low overall risk of bias, while the remaining studies exhibited a moderate risk, mainly due to confounding factors and participant selection bias (Figure 2). Retrospective study designs in some cases introduced selection bias, as patients were not randomly allocated, and missing data in studies with longer follow-ups further impacted result reliability. Studies by Hellekant et al. (1989) [15], Janson et al. (2012) [19], Gorucu-Coskuner et al. (2017) [22], Mahmoudzadeh et al. (2018) [23], and Bhupali et al. (2019) [24] had a moderate confounding bias, indicating that factors such as patient compliance and treatment variations could have influenced outcomes. Bias due to missing data was particularly noted in longer follow-up studies, potentially introducing attrition bias. However, the classification of interventions and deviations from intended treatments were consistently low across studies, ensuring well-defined treatment protocols, though subjective assessment methods led to moderate measurement bias in some cases. Overall, while the studies included in this review provide valuable insights into the comparison between extraction and non-extraction treatments, the presence of moderate bias in several domains underscores the need for future research with more standardized methodological approaches, including randomized controlled trials and standardized outcome measures to enhance the reliability of findings.

Certainty of evidence across the included studies

The rating of various domains when assessing the certainty of evidence across the different articles included in the present systematic review using the GRADE (Grading of Recommendations, Assessment, Development, and Evaluation) approach is summarized in Table 2.

**Table 2:** Rating the various domains of evidence quality using the GRADE approach GRADE, Grading of Recommendations, Assessment, Development, and Evaluation

Author (Year)	Risk of Bias	Inconsistency	Indirectness	Imprecision	Publication Bias	Overall Quality
Hellekant et al. (1989)	Moderate	Not serious	Not serious	Moderate	Uncertain	Moderate
Bishara et al. (1994)	Moderate	Not serious	Not serious	Not serious	Uncertain	Moderate
Rossouw et al. (1999)	Not serious	Not serious	Not serious	Not serious	Uncertain	High
Erdinc et al. (2006)	Not serious	Not serious	Not serious	Not serious	Uncertain	High
Janson et al. (2012)	Not serious	Not serious	Not serious	Not serious	Uncertain	High
Zafarmand et al. (2014)	Not serious	Not serious	Not serious	Not serious	Uncertain	High
Francisconi et al. (2014)	Not serious	Not serious	Not serious	Not serious	Uncertain	High
Gorucu-Coskuner et al. (2017)	Not serious	Not serious	Not serious	Not serious	Uncertain	High
Khosravi et al. (2018)	Not serious	Not serious	Not serious	Not serious	Uncertain	High
Bhupali et al. (2019)	Not serious	Not serious	Not serious	Not serious	Uncertain	High

The body of evidence supporting the comparison between extraction and non-extraction treatment outcomes is largely robust, with most studies demonstrating high-quality evidence based on the GRADE criteria. In particular, the more recent investigations by Rossouw et al. (1999), Erdinc et al. (2006), Janson et al. (2012), Zafarmand et al. (2014), Francisconi et al. (2014), Gorucu-Coskuner et al. (2017), Khosravi et al. (2018), and Bhupali et al. (2019) exhibit minimal concerns regarding risk of bias, inconsistency, indirectness, and imprecision. These studies provide consistent and precise estimates that directly address the clinical question of post-treatment relapse, reflecting improved study designs and methodological rigor. Although publication bias remains uncertain, the strength and consistency of the findings from these later studies underpin a high level of confidence in the overall evidence.

In contrast, earlier studies such as Hellekant et al. (1989) and Bishara et al. (1994) are rated as moderate quality due to modest concerns regarding risk of bias and imprecision, limitations that are likely related to smaller sample sizes and less rigorous control of confounding factors. Despite these limitations, the contributions of Hellekant et al. (1989) and Bishara et al. (1994) remain valuable in providing historical context and broadening the overall evidence base. Taken together, while acknowledging the moderate quality of the earliest evidence, the cumulative findings from the systematic review indicate that the more recent research offers robust and reliable data to inform clinical decision-making regarding extraction versus non-extraction treatment protocols.

Synthesis of results

The meta-analysis was conducted with an SMD as a summary statistic measure for comparison between extraction and non-extraction groups to assess the outcomes in terms of overjet, overbite, and Little's Irregularity Index.

Overjet

**Figure 3:**
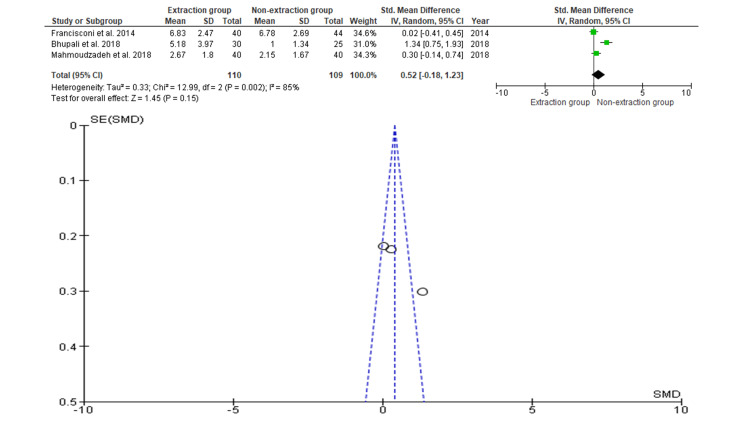
Comparison of overjet relapse between the extraction and non-extraction groups and publication bias, indicated by Begg's funnel plot with 95% CI SMD, standardized mean difference

Three studies contained data on 219 participants, of which 110 participants were evaluated by extraction and 109 patients were evaluated by non-extraction for the evaluation in terms of overjet [21,23,24]. As shown in Figure 3, the SMD is 0.52 (-0.18 - 1.23), and the pooled estimates favor the non-extraction group, but this difference is not statistically significant (p>0.05). The funnel plot did not show significant asymmetry, indicating an absence of publication bias.

Overbite

**Figure 4:**
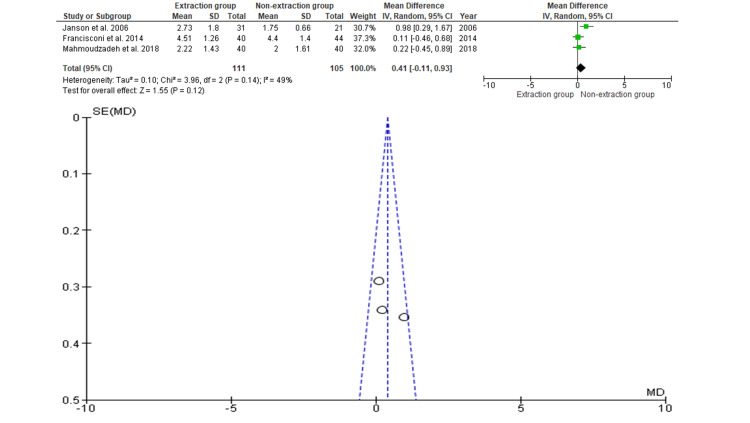
Comparison of overbite relapse between the extraction and non-extraction groups and publication bias, as indicated by Begg’s funnel plot with a 95% CI

Three studies with data on 216 participants were included, of which 111 participants were evaluated with extraction and 105 participants were evaluated with non-extraction for the assessment of overbite [19,21,23]. As shown in Figure 4, the SMD is 0.41 (-0.11 to 0.93), and the pooled estimates favor the non-extraction group, but this difference is not statistically significant (p>0.05). The funnel plot did not show significant asymmetry, indicating an absence of publication bias.

Little’s Irregularity Index

**Figure 5:**
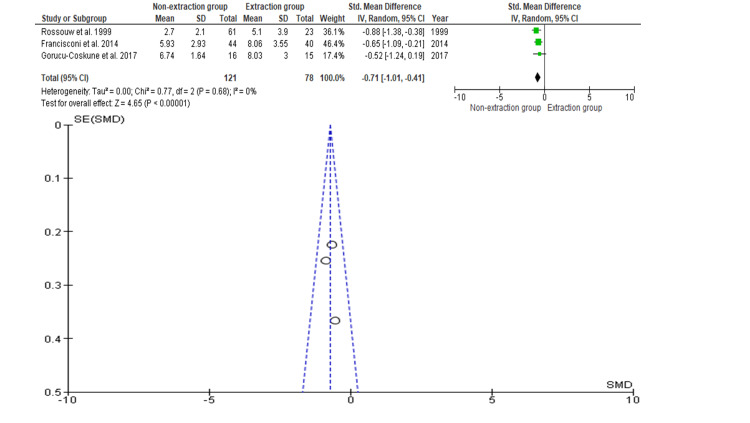
Comparison of Little’s Irregularity Index between the extraction and non-extraction groups and publication bias, as indicated by Begg’s funnel plot with a 95% CI

Three studies with data on 199 participants were included, of which 121 participants were evaluated with extraction and 78 participants were evaluated with non-extraction for the assessment of the Little's Irregularity Index. As shown in Figure 5 [17,21,22], the SMD is 0.71 (-0.01 to 1.23), and the pooled estimates favor the non-extraction group. This suggests that, on average, overjet is 0.52 times greater in the non-extraction group compared to the extraction group, but this difference is not statistically significant (p>0.05). The funnel plot did not show significant asymmetry, indicating an absence of publication bias.

Discussion

The results of this systematic review and meta-analysis suggest that both extraction and non-extraction orthodontic treatments achieve comparable long-term stability in terms of overjet, overbite, and incisor irregularity. Although the pooled estimates for overjet and overbite relapse favored the non-extraction group, the differences were not statistically significant, indicating that both treatment approaches can provide satisfactory outcomes with appropriate retention protocols. The slight advantage observed in the non-extraction group could be attributed to the preservation of arch length and the avoidance of potential biomechanical effects associated with space closure in extraction cases. However, the clinical significance of these differences remains uncertain, and the decision between extraction and non-extraction should be based on individual patient needs and occlusal considerations.

The findings related to Little's Irregularity Index indicate a trend of higher relapse rates in the non-extraction group, which aligns with the understanding that untreated crowding has a greater tendency to relapse over time. Non-extraction treatments often rely on transverse expansion and incisor proclination to achieve alignment, both of which can contribute to long-term instability due to soft tissue pressures and natural aging processes [25,26]. In contrast, extraction cases provide space for proper tooth alignment and allow for more stable arch form maintenance, which may explain the relatively lower irregularity index relapse observed in those cases. This highlights the importance of meticulous space management and long-term follow-up in non-extraction treatments to prevent potential relapse.

Despite the absence of significant publication bias in the included studies, some degree of heterogeneity was noted across the studies in terms of sample size, follow-up duration, and retention strategies. These variations could influence the reported outcomes and should be considered when interpreting the findings. Factors such as patient compliance with retention protocols, biological variability in response to treatment, and differences in orthodontic mechanics employed may have contributed to the observed inconsistencies [27,28]. Future studies with standardized retention protocols and longer follow-up durations are warranted to provide more conclusive evidence regarding treatment stability.

From a clinical perspective, the findings underscore the importance of individualized treatment planning that considers not only the initial malocclusion but also long-term stability factors such as arch form preservation, periodontal health, and patient compliance. While non-extraction treatment may be preferable in cases where facial profile and soft tissue harmony are a primary concern, extraction therapy should not be overlooked when space management is critical to achieving stable occlusion. Additionally, retention strategies should be customized based on the treatment modality, with non-extraction patients potentially requiring more prolonged retention to mitigate relapse risks.

The limitations of the included studies, such as retrospective designs and varying assessment methods, highlight the need for future prospective randomized controlled trials to provide stronger evidence on the long-term outcomes of extraction versus non-extraction treatments. Additionally, the role of adjunctive measures such as interproximal reduction and fixed retainers in enhancing stability should be explored to further optimize post-treatment outcomes. Overall, while the studies included in this review provide valuable insights into the comparison between extraction and non-extraction treatments, the presence of moderate bias in several domains reinforces the need for future research with more standardized methodological approaches. There is a particular need for conducting randomized controlled trials and standardized outcome measures to enhance the reliability of findings. While both treatment modalities can achieve satisfactory long-term stability, careful patient selection and adherence to retention protocols are crucial to maintaining treatment outcomes and minimizing relapse risks.

## Conclusions

The findings of this systematic review and meta-analysis suggest that there is no statistically significant difference in the relapse of overjet, overbite, and Little's Irregularity Index between extraction and non-extraction treatment modalities. Although the pooled estimates favor the non-extraction group in all evaluated parameters, the differences observed were not clinically significant, indicating that both treatment approaches can achieve comparable long-term stability. The variability in relapse outcomes across studies highlights the influence of individual patient factors, treatment techniques, and retention protocols on orthodontic stability. Despite the moderate risk of bias in several included studies, the overall evidence suggests that clinicians should focus on individualized treatment planning based on patient-specific needs rather than a generalized preference for extraction or non-extraction. Future well-designed longitudinal studies with standardized outcome measures and longer follow-up periods are necessary to further validate these findings and provide more definitive clinical recommendations.
